# Intracellular Relaxometry, Challenges, and Future Directions

**DOI:** 10.1021/acscentsci.2c00976

**Published:** 2022-10-20

**Authors:** Alina Sigaeva, Neda Norouzi, Romana Schirhagl

**Affiliations:** University Medical Center Groningen, University of Groningen, Antonius Deusinglaan 1, 9713AV Groningen, The Netherlands

## Abstract

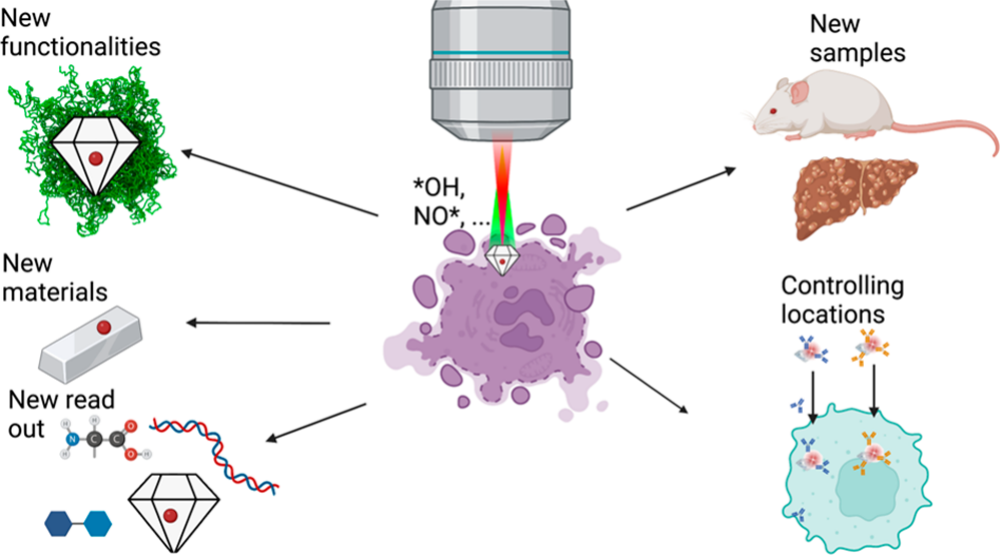

Nitrogen vacancy (NV) centers change their optical properties on the basis of their magnetic surroundings. Since optical signals can be detected more sensitively than small magnetic signals, this technique allows unprecedented sensitivity. Recently, NV center-based relaxometry has been used for measurements in living cells with subcellular resolution. The aim of this Outlook is to identify challenges in the field, including controlling the location of sensing particles, limitations in reproducibility, and issues arising from biocompatibility. We further provide an outlook and point to new directions in the field. These include new diamond materials with NV centers, other defects, or even entirely new materials that might replace diamonds. We further discuss new and more challenging samples, such as tissues or even entire organisms, that might be investigated with NV centers. Then, we address future challenges that have to be resolved in order to achieve this goal. Finally, we discuss new quantities that could be measured with NV centers in the future.

## Abstract 2

Recently, quantum sensing in living cells has been demonstrated. This Outlook discusses the current challenges of this field as well as future directions we expect the field will take.

## 1 Introduction

Beyond their use in jewelry, diamonds are used as abrasives and drilling materials for their hardness. Protected within an inert crystal lattice, specific defects are present or can be engineered.^1^ These so-called color centers do not bleach or blink and are infinitely photostable.^2^ Hundreds of color centers are described in diamonds.^3^ The nitrogen vacancy (NV) defect, the most studied type, consists of a nitrogen atom with a vacancy next to it. NV centers exhibit bright fluorescence from 550 to 800 nm.^2,4^ Nanodiamonds (NDs) with these color centers are attractive for long-term fluorescent imaging as well as super-resolution microscopy.^5,6^ NV centers usually exist in neutral NV^0^ and negatively charged NV^–^ forms.^7^ The NV^–^ is used in quantum-sensing applications to detect external electromagnetic fields on the basis of the optical readout. The fluorescence intensity is dependent on its magnetic environment. Since fluorescence detection is very sensitive, this method even allows one to record the signals of single electrons or a few nuclei.^8−10^

NV centers allow one to investigate magnetic nanostructures, domain walls in magnetic structures,^11^ paramagnetic ions in solution,^12^ spin-labeled molecules, or proteins with metallic parts.^13^ They can also be used to increase the sensitivity of biosensors.^14^ Since their magnetic resonance is very temperature sensitive, NV centers can be used to detect temperature changes in the milli-Kelvin range with nanoscale resolution.^15−18^ Recently, NV centers have been used for measurements in cells. Davis et al.^19^ imaged spin-labeled slices of fixed cells, while McGuinness et al. measured the orientation of a particle within a cell.^20,21^ Later, even measurements of metabolic activity have been achieved in live yeast cells,^22^ immune cells,^23^ and sperm cells,^24^ during the particle’s transport inside the cell,^22^ or during viral infection.^26^ Recently, the first relaxometry measurements in primary cells from donors have been demonstrated.^27^ Here, we discuss the current limitations of this method and the future direction this exciting field might take.

## 2 The Relaxometry Principle

**Figure 1:**
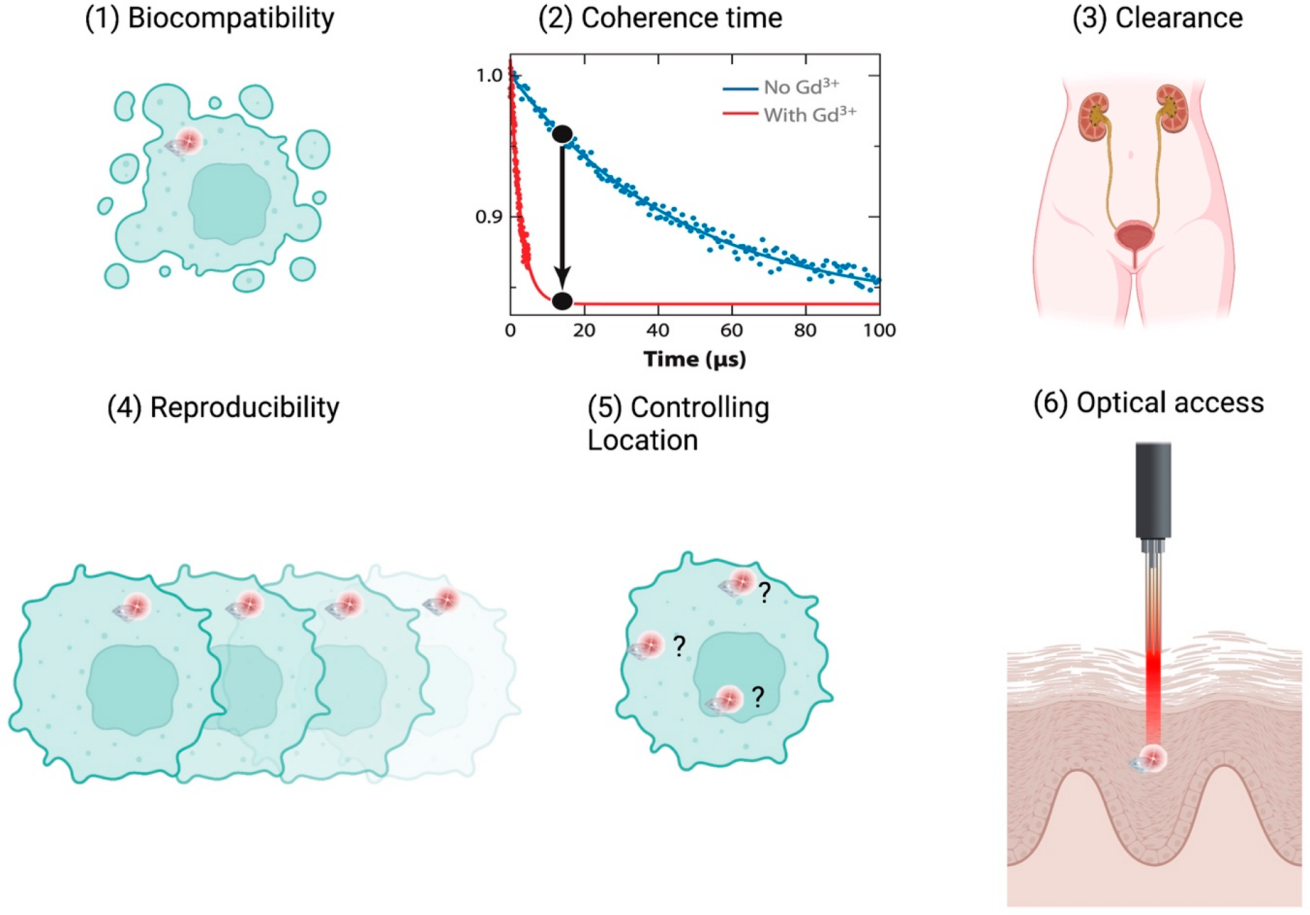
Challenges for intracellular quantum sensing: (1) While biocompatibility is generally excellent, there are limitations for using large concentrations of NDs. (2) Coherence times limit the sensing performance. (3) A major challenge for *in vivo* sensing is the lack of clearance from the body. (4) While for applications in physics it is often possible to reuse a good NV center, this is often not possible in a biomedical setting. As a result, it is imperative that measurements with different NV centers are reproducible. (5) For many applications, it is crucial to control where the measurement takes place in the cell. (6) In order to collect an optical signal from a diamond, one needs optical access. This is a serious challenge for thick, pigmented, or highly refractive samples. The application that is shown is a measurement in a skin tissue or on the intact skin of a patient.

The NV center has three electronic states available to its six electrons: a triplet ground state, a triplet excited state, and a singlet metastable state. Each of the triplet states, in turn, has three spin sublevels: the degenerate *m*_S_ = ± 1 sublevels and the *m*_S_ = 0 sublevel. By default, the electrons are in a thermal equilibrium between the *m*_S_ = ± 1 and the *m*_S_ = 0 sublevels of the ground state. In a relaxometry experiment, the NV centers are first pumped into the bright *m*_S_ = 0 state and then left to relax into the (darker) natural stochastic combination of *m*_S_ = 0 and *m*_S_ = ± 1. The relaxation happens faster in the presence of external magnetic noise from unpaired electrons of free radicals or spin labels (see Figure 1(2)). The current state of the NV center can be read out by its fluorescence intensity. This approach allows one to perform the sensing, using only the optical means. It can be implemented in a confocal microscope-like setup if one can make the excitation laser pulse.

## 3 Current Limitations and Future Prospects

**Figure 2:**
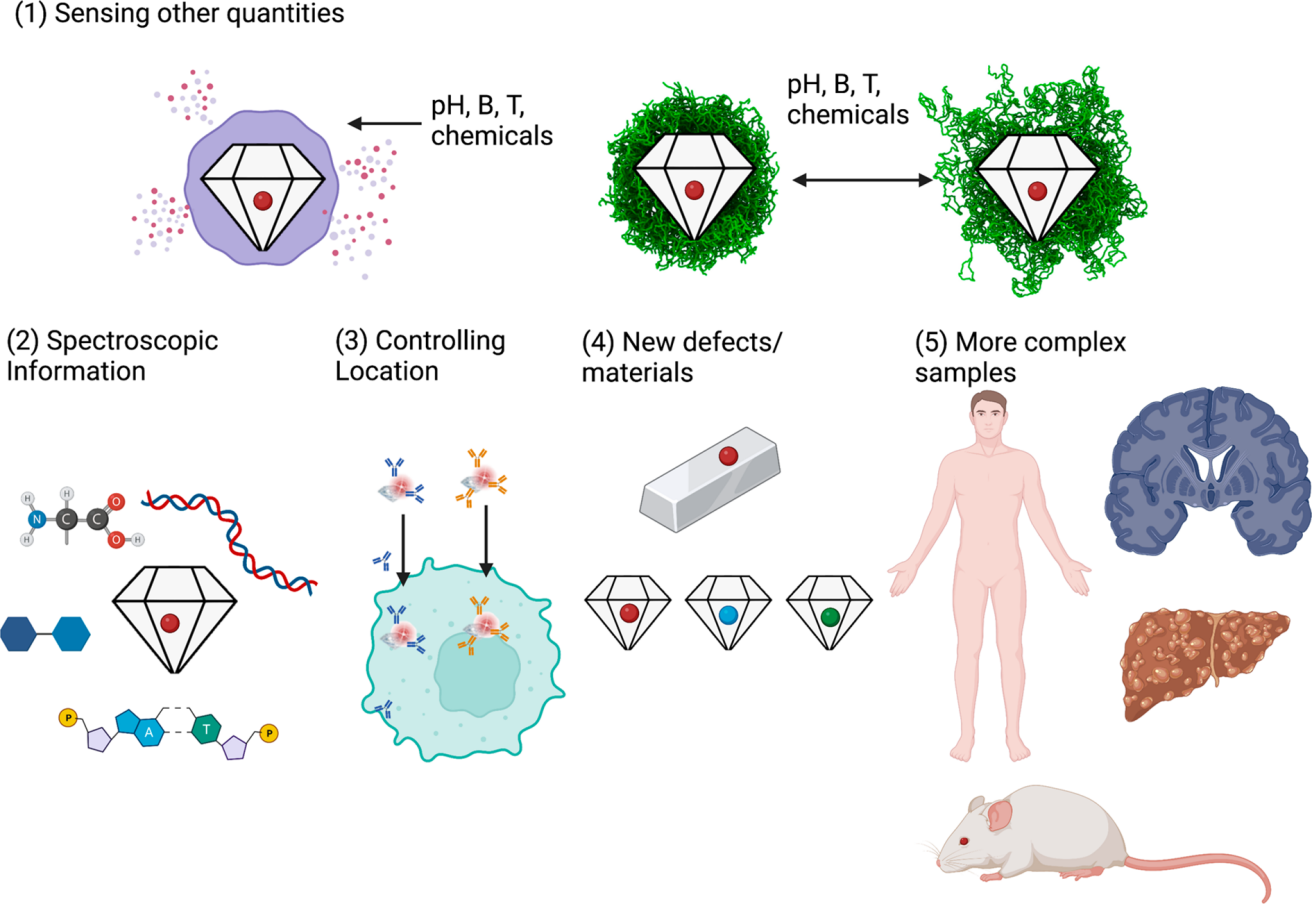
Future directions. (1) To detect quantities other than electromagnetic fields, NDs can be combined with other responsive materials. A responsive shell can release spin labels after a stimulus, inducing the change in *T*_1_. Alternatively, particles can have a coating that expands or shrinks depending on the stimulus, moving the spin labels closer to or further away from the NV center. (2) Using more complex pulsing sequences, including for instance double electron–electron resonance or the NV center equivalent of a nuclear magnetic resonance measurement, it might be possible to differentiate radicals and nuclei and preform intracellular spectroscopy. (3) One challenge is to control the location of the measurement. (4) New types of defects or even replacing diamonds with different materials might lead to an improved sensing performance. (5) More complex samples might be of interest, including isolated tissues, organs, animals, or even humans.

A summary of future prospects that we expect is given in Figure 2.

### 3.1 Other Defects or Materials

So far, only NV centers have been used for quantum sensing in living cells. Currently, one of the biggest challenges of using NV centers is reproducibility from one particle to the next. One way to achieve this goal is to use large ensembles of NV centers.^12^ However, since it is a necessity that the NV centers are close to the diamond surface and thus to the sample, the number of NV centers that can be used is limited. At the same time, there are a few color centers that have been proven useful in physics.^28,29^ However, in biology, they need to operate at room temperature and be stable in NDs in a high enough concentration. The latter problem might be resolved in the future. Due to the autofluorescence of biosamples, it is preferable to have emission in the near-infrared region, the so-called biological window. Besides defects in diamonds, also entirely different materials, might emerge in the future, potentially hosting color centers. Interesting candidates are other wide bandgap semiconductors like silicon carbide^30^ or gallium arsenide.^31^ Also, molecular systems might prove to be valuable.^32^

### 3.2 ND Internalization into Cells

Fortunately, NDs are exceptionally biocompatible in cells,^33,34^ in animal testing, and even in humans.^11,12^ Many cells can easily take up nanoparticles via different pathways.^35−40^ By functionalizing the surface of NDs, it is also possible to change the uptake pathway and efficiency.^41^ The uptake is usually impeded, if the cell is small (bacterial cells) or has a pronounced cell wall (bacterial, yeast, plant cells).^42^ Certain mammalian cells (e.g., neurons or some epithelial cells^43^) also do not internalize nanoparticles easily.^36,44,45^ The uptake can be forced by more invasive procedures such as electroporation, microinjection, and chemical transformation. While these approaches generally increase the uptake rates, they can harm the cells. Moreover, the intracellular fate of NDs can differ, depending on the exact internalization procedure.

### 3.3 Controlling the Intracellular Location

The full potential of the technique can only be used if we know or even control where NDs are within cells. Internalized NDs are generally encapsulated in intracellular vesicles: endosomes or phagosomes. These vesicles eventually fuse with lysosomes, and the majority of NDs ultimately exit the vesicles through endosomal escape,^28^ generally not entering the nucleus or other organelles. The timing and efficiency of the endosomal escape depends on the cell type^46^ and shape of the NDs, among other factors.^47,48^ “Prickly” NDs have a higher chance of escaping the endosomes. Another approach to increase endosomal escape is to functionalize the ND surface with cationic polymers causing rupture of the vesicles and releasing the cargo into the cytoplasm.^49^ Lastly, the vesicular compartment can be bypassed using nonendocytic uptake protocols (electroporation, microinjections, chemical transformation).^42^ In certain cases, one might want retainment of the NDs in the vesicular compartment.^50^ More rounded as well as larger ND particles can increase the endosomal retainment.

A number of approaches have been used to achieve targeting of the NDs^34,51^ with intracellular localization sequences^52,53^ or antibodies.^20,54^ One challenge is to preserve the functionality of the targeting moiety, as it is exposed to the complex mixture of proteins and salts, changing pH of the endolysosomes, and intracellular enzymes.

A different approach is to deduct the properties of the ND’s environment from the way the particles moved during the experiment. It is possible to combine single-particle tracking and trajectory analysis with *T*_1_ measurements to get a map of *T*_1_ values as the ND moves through the cell.^25^ This approach requires a complicated analysis of the trajectories, as NDs move in complex patterns through a highly nonuniform intracellular environment.

### 3.4 More Complex Samples

While measurements in isolated living cells have been successfully performed, there are other interesting, more complex samples. While in cultured cells optical access is easily achieved, this is challenging in thick tissues or large organisms. In *ex vivo* studies, individual cells can be extracted from a tissue sample and cultured.^55^ However, many processes can only be studied when cells are in their biological context. These include biodistribution, clearance by the body, or certain processes in disease progression. A further challenge is to know where the diamond particle is within a complex sample (e.g., in the cell of which type) and to provide the biological context for the measurements. To study some interesting phenomena, one might need to measure deep within the body. One solution is to provide optical access via an optical fiber with NDs or macroscopic diamonds attached to it.^56^ These fibers could then be inserted into the body similar to an endoscopic device to both provide the excitation laser beam and collect fluorescent signals from the diamond. These kinds of measurements are limited to organs that can be accessed with endoscopy like the intestinal tract or the lungs.

Additionally, more complex biological samples are often more fragile and must be measured while they are as close to their physiological state as possible. One should therefore prevent long transport between laboratories. Ideally, biological or medical laboratories must be equipped with commercial magnetometry equipment.

### 3.5 Measuring Other Quantities

There is a number of interesting parameters that might be detected with quantum sensing. Applying responsive coatings, where for instance a spin label is detached or moved further away from the NV center, offers a way to extend the usefulness of relaxometry, for instance, to measure pH^57^ or to detect biomarkers.^58^

Using more complex pulsing sequences than relaxometry, it might be possible to obtain spectral information on the electrons or even nuclei surrounding the nanodiamonds.^59^ One of these pulsing sequences is the double electron–electron resonance or DEER sequence,^60^ which can differentiate between radicals or other sequences that can differentiate^59^ between nuclei and resolving chemical shifts.^61^ However, a major challenge of the realization of this application is that the particles are moving and rotating, and currently, available nanodiamonds have relatively short coherence times, which limit the sensing performance.

Another attractive avenue is to complement quantum sensing with other techniques. While this is already done with optical microscopy, there might be opportunities to combine relaxometry with other techniques, which allow single-cell resolution, such as single-cell omics.

## 4 Conclusions

Relaxometry is a versatile technique with distinct advantages including nanoscale resolution, real-time measurements, and sensitivity for free radicals. The method is also all-optical and thus relatively straightforward to implement. In the future, we anticipate new directions in the field, including an extension to more complex samples. We expect that in the future relaxometry data will be further correlated with other biological information as well as data from other single-cell techniques.
